# Comparative Immunogenicity of COVID-19 Vaccines in a Population-Based Cohort Study with SARS-CoV-2-Infected and Uninfected Participants

**DOI:** 10.3390/vaccines10020324

**Published:** 2022-02-18

**Authors:** David Peterhoff, Sebastian Einhauser, Stephanie Beileke, Hans-Helmut Niller, Felix Günther, Michael Schachtner, Benedikt Asbach, Philipp Steininger, Matthias Tenbusch, Antonia S. Peter, Andre Gessner, Ralph Burkhardt, Iris M. Heid, Ralf Wagner, Klaus Überla

**Affiliations:** 1 Institute of Medical Microbiology and Hygiene, Molecular Microbiology (Virology), University of Regensburg, Franz-Josef-Strauß-Allee 11, 93053 Regensburg, Germany; david.peterhoff@klinik.uni-regensburg.de (D.P.); sebastian.einhauser@klinik.uni-regensburg.de (S.E.); hans-helmut.niller@klinik.uni-regensburg.de (H.-H.N.); michael.schachtner@klinik.uni-regensburg.de (M.S.); andre.gessner@klinik.uni-regensburg.de (A.G.); 2 Institute of Clinical Microbiology and Hygiene, University Hospital Regensburg, Franz-Josef-Strauß-Allee 11, 93053 Regensburg, Germany; 3 Institute of Clinical and Molecular Virology, University Hospital Erlangen, Friedrich-Alexander Universität Erlangen-Nürnberg, Schlossgarten 4, 91054 Erlangen, Germany; stephanie.beileke@uk-erlangen.de (S.B.); philipp.steininger@uk-erlangen.de (P.S.); matthias.tenbusch@fau.de (M.T.); antonia.peter@fau.de (A.S.P.); 4 Department of Mathematics, Stockholm University, Kräftriket 6, 106 91 Stockholm, Sweden; felix.gunther@math.su.se; 5 Department of Genetic Epidemiology, University of Regensburg, Franz-Josef-Strauß-Allee 11, 93053 Regensburg, Germany; iris.heid@ur.de; 6 Institute of Clinical Chemistry and Laboratory Medicine, University Hospital Regensburg, Franz-Josef-Strauß-Allee 11, 93053 Regensburg, Germany; ralph.burkhardt@klinik.uni-regensburg.de

**Keywords:** SARS-CoV-2, vaccination, population-based cohort, immunogenicity, neutralizing antibodies, Comirnaty, Spikevax, Vaxzevria, variants of concern VoC

## Abstract

To assess vaccine immunogenicity in non-infected and previously infected individuals in a real-world scenario, SARS-CoV-2 antibody responses were determined during follow-up 2 (April 2021) of the population-based Tirschenreuth COVID-19 cohort study comprising 3378 inhabitants of the Tirschenreuth county aged 14 years or older. Seronegative participants vaccinated once with Vaxzevria, Comirnaty, or Spikevax had median neutralizing antibody titers ranging from ID50 = 25 to 75. Individuals with two immunizations with Comirnaty or Spikevax had higher median ID50s (of 253 and 554, respectively). Regression analysis indicated that both increased age and increased time since vaccination independently decreased RBD binding and neutralizing antibody levels. Unvaccinated participants with detectable N-antibodies at baseline (June 2020) revealed a median ID50 of 72 at the April 2021 follow-up. Previously infected participants that received one dose of Vaxzevria or Comirnaty had median ID50 to 929 and 2502, respectively. Individuals with a second dose of Comirnaty given in a three-week interval after the first dose did not have higher median antibody levels than individuals with one dose. Prior infection also primed for high systemic IgA levels in response to one dose of Comirnaty that exceeded IgA levels observed after two doses of Comirnaty in previously uninfected participants. Neutralizing antibody levels targeting the spike protein of Beta and Delta variants were diminished compared to the wild type in vaccinated and infected participants.

## 1. Introduction

Currently, there are four widely used COVID-19 vaccines in Europe and their immunogenicity has been assessed during clinical development [1,2,3] and post-marketing follow-up studies [4,5,6,7]. Different study populations, assays, and time points of analysis hinder direct comparisons between the different vaccines. Investigations on the immune responses after mix-and-match regimens also included conventional homologous immunizations enabling comparisons between different vaccines, but are limited to time points of analyses immediately after the second immunization and particular study populations younger than 60 years of age [8,9,10,11]. We therefore determined antibody responses to different vaccines in a population-based cohort under real-world conditions. The cohort was established in June 2020 to assess seroprevalence for SARS-CoV-2 antibodies in the county of Tirschenreuth [12], which was one of the hardest-hit regions in Germany early during the pandemic. The 4201 participants were aged 14 years and older and were representative of the county’s population [12]. Two follow-up surveys including serum sampling and a questionnaire on the participants’ SARS-CoV-2 infection and vaccination history were performed in November 2020 and April 2021 with 3546 and 3391 participants, respectively. Seroprevalence data from the November 2020 and April 2021 sampling revealed a cumulative N-antibody seropositivity of 8.93% and 13.30%, respectively (Einhauser et al., manuscript in preparation). Since the vaccination roll-out in Germany started at the end of December 2020, the April 2021 samples allowed to assess the antibody responses after vaccination and to compare them to those induced by infection or immunization after infection. Dependence of the antibody responses on the age of vaccinees and on the time since the second immunization were also of interest considering the current need for booster immunizations.

## 2. Materials and Methods

### 2.1. Cohort

The cohort at baseline was described thoroughly in Wagner et al. 2021. In brief, 6540 of 6608 randomly selected inhabitants of Tirschenreuth aged 14 years or older were invited to participate in the TiKoCo study in June 2020. Overall, 4203 individuals participated between 28 June and 13 July 2020 by giving 5.7 mL blood and filling out a questionnaire, yielding a net response of 64.26%, with a higher response among the age group 20–74 years compared to those 14–19 years or 75+ years of age and slight differences amongst the 26 local municipalities. The 4203 participants included 48.3% men; age ranged from 14 to 102 years (refer to Table 1 in Wagner et al. [12] for more detailed information). Of the 4203 participants at baseline, 3534 successfully participated in the first follow-up between 16 November and 27 November 2020, while 3378 participated in the second follow-up (between 19 April and 30 April 2021), yielding a follow-up response of 84.1 and 80.4%, respectively. Overall, 3196 participants took part in all three rounds of investigation, leading to a full participation rate of 76.0%. (Einhauser et al. manuscript in preparation). For vaccination, self-reports on vaccination status, type of vaccine, and date of vaccination were obtained by questionnaire at T3. Age at baseline was determined by data from population registries and the time since vaccination was determined from the time between follow-up 2 and the reported date of vaccination.

The TiKoCo study was approved by the Ethics Committee of the University of Regensburg, Germany (vote 12-101-0258) and adopted by the Ethics Committee of the University of Erlangen (vote 248_20 Bc). The study complies with the 1964 Helsinki declaration and its later amendments. All participants provided written informed consent.

### 2.2. Antibody Assays

Participants with prior SARS-CoV-2 infection were identified by the detection of antibodies to nucleoprotein N. The Elecsys Anti-SARS-CoV-2 N test (Roche Diagnostics GmbH, Mannheim, Germany) [13] detecting all classes of immunoglobulins directed to the nucleoprotein N was operated on the COBAS pro e 801 module according to the manufacturer’s recommendations. Cutoff values were chosen as specified by the manufacturer. Participants without detectable levels of N antibodies at all study time points were considered uninfected and assigned the N antibody status T0. Participants seropositive for N antibodies at baseline (June 2020) were assigned the N-antibody status T1. T3 participants were seronegative at baseline and at the follow-up 1 (November 2020) but converted to detectable N antibody levels at follow-up 2 (April 2021).

To assess protective antibody responses after infection and immunization, four different assays were performed: (i) The Elecsys Anti-SARS-CoV-2 S test (Roche Diagnostics GmbH, Mannheim, Germany) [13] detecting complete Ig directed to spike (S) protein receptor-binding domain (RBD) was operated on the COBAS pro e 801 module according to the manufacturer’s recommendations. Cutoff values were chosen as specified by the manufacturer. According to information provided by Roche [13,14], 1 U/mL (Elecsys) corresponds to 1 BAU/mL (WHO standard). (ii) Our validated in-house ELISA detecting IgA antibody responses to the SARS-CoV-2 spike protein’s receptor-binding domain (RBD) was performed as described earlier [15]. (iii) The neutralization assay using the Vesicular Stomatitis Virus (VSV-Δ G*FLuc) pseudotyped with wt-SARS-CoV-2-Spike-ΔER was performed as described earlier [14]. In brief, an inoculum of 25,000 ffu was neutralized with a 2-fold serum dilution series for 1 h, and luciferase activity was determined 20 h post infection of HEK293T-ACE2 + -cells using BrightGlo (Promega Corp, Madison, WI, USA). iv) The capacity of antisera to neutralize different VoCs was determined using a lentiviral pseudotype assay. As described previously [16,17], lentiviral vector particles expressing luciferase and pseudotyped with the spike protein of the different VOCs were produced on HEK 293T cells (ECACC 12022001). The neutralization activity of serial dilutions of sera was analyzed on a HEK293T cell line overexpressing-ACE2 48 h after infection [18]. The cells were exposed to a lentiviral pseudotype dose resulting in 2.5 − 10 × 10^4^ RLU/s in the absence of serum. The 50% inhibitory dilution (ID50) of the sera was calculated with Prism 9 GraphPad (San Diego, CA, USA).

### 2.3. Statistical Analysis

We compared antibody responses in the different vaccine groups and groups of participants with different infection statuses by non-parametric tests. For each research question regarding two groups of independent individuals, we applied a Mann–Whitney test. For questions regarding more than two groups of independent individuals, a Kruskal–Wallis test for the overall group comparison was performed just followed by a Mann–Whitney test for pairwise comparisons. Significance was judged at 0.05, except for pairwise tests implying multiple testing where a Bonferroni-corrected level of 0.05 divided by the number of tests was applied. For comparisons including the same individuals comparing different measurements, we applied the Wilcoxon rank-sum test. These analyses were performed with Prism 9 GraphPad.

### 2.4. Regression Analysis

We used generalized additive regression to jointly investigate the association of quantitative antibody levels with time after vaccination and age (measured at baseline) among study participants vaccinated twice with Comirnaty. We estimated a separate model for RBD binding and neutralizing antibodies (ID_50_). For each, we modeled the quantitative antibody levels in a Gaussian model with log-link (assumption of a conditional log-normal distribution for the quantitative antibodies) and estimated non-linear associations with the mean using a thin plate regression spline per covariate. Smoothing parameter selection was based on restricted maximum likelihood (REML). To investigate the possibility of differing trajectories of antibody decrease with time for individuals of varying age, we also estimated a bivariate spline-surface and compared model fit based on the explained deviance. We illustrated the model results by inspecting the estimated (non-linear) associations and visualizing the predicted antibody levels over the range of observed times after vaccination (time between second vaccination and blood drawn at the second follow-up, 14–100 days) for an age of 30, 55, or 80 years, respectively. The models were estimated using the mgcv-package in R [19,20].

For comparisons of proportions, two-sided Fisher’s exact tests were performed by an online tool at https://www.langsrud.com/stat/fisher.htm (accessed on 21 December 2021). *p*-Values were judged at Bonferroni-corrected significance level when multiple testing was implied.

## 3. Results

### 3.1. Antibody Responses Induced by the Various Vaccines among Previously Infected Individuals

The antibody response after infection with SARS-CoV-2 was determined as a benchmark for the immunogenicity of vaccines. In sera from the April 2021 visit (T3), we evaluated the neutralizing and the spike protein RBD binding antibody levels (Roche Elecsys Anti-SARS-CoV-2 S test) after infection in individuals that were unvaccinated as well as in individuals that were vaccinated.

**Figure 1:**
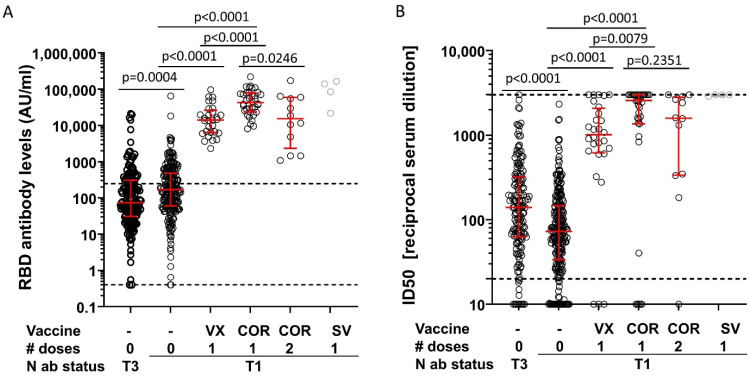
Antibody responses in infected participants. Shown are antibody levels to the receptor-binding domain (RBD) of the S protein (Roche Elecsys Anti-SARS-CoV-2 S test; arbitrary units AU) (**A**) and neutralizing antibody levels (ID50) (**B**) in seropositive participants receiving the indicated number of doses of Vaxzevria (VX), Comirnaty (COR), or SpikeVax (SV). Median and interquartile range are indicated by red bars. The Kruskal–Wallis test including all five groups with more than six participants indicated significant differences for the RBD and neutralizing antibody levels (*p* < 0.0001 for both, judged at 5% significance level). Pairwise comparisons were performed to (i) analyze a potential waning of immune responses after infection (0 dose T3 vs. 0 dose T1), (ii) determine and compare the effect of one dose of Vaxzevria and Corminaty (0 dose T1 vs. 1 dose VX vs. 1 dose COR) and (iii) explore the effect of a second dose of Cormirnaty (1 dose COR vs. 2 dose COR). *p*-values of these pairwise group comparisons with the Mann–Whitney test are shown (judged for significance at a Bonferroni-corrected level of 0.05/5 = 0.01 to account for 5 tests). N antibody (ab) status: T1: participants that were N-antibody positive already at the June 2020 visit; T3: participants negative for N antibodies at the June and November 2020 visits, but positive at the April 2021 visit. Dashed lines in (**A**) mark the upper and lower limits of the linear range of the assay, the dashed line in (**B**) mark the upper and lower limits of detection. Supplementary Tables S1 and S2 provide number of participants, basic demographic characteristics and the median time interval between the last immunization and blood sampling. # doses indicates the number of applied doses.

First, we compared the sub-cohort of unvaccinated participants that were N-antibody positive already at the June 2020 visit (T1, time to blood drawn at T3 > 9 months, *n* = 227) with the sub-cohort of unvaccinated participants that seroconverted for N antibodies between the November 2020 (T2) and the April 2021 visit (T3, *n* = 164). This recent seroconverter group had lower RBD binding antibody levels but higher neutralizing antibody levels than the sub-cohort that had been infected at least 9 months before blood sampling (T3 versus T1) (Figure 1A,B; RBD median = 73 versus 171; ID_50_ median = 140 versus 72). Of note, participants of the sub-cohort infected >9 months ago may include a small percentage of individuals re-infected within the last 9 months before blood drawn at T3.

Second, to assess antibody response after vaccination in previously infected participants, we restricted the analysis to participants that were N-antibody positive at the June 2020 visit (T1), since this excluded the possibility that participants were immunized prior to a SARS-CoV-2 infection. The RBD and neutralizing antibody levels were then compared between those that were not vaccinated and those receiving one vaccination with Vaxzevria or Comirnaty. Demographic characteristics for the different sub-cohorts, sample size by group, and median time between last immunization and blood sampling are summarized in Supplementary Tables S1 and S2. Clearly, a single immunization with Vaxzevria or Comirnaty strongly enhanced the median RBD antibody levels even above the upper limit of the linear range of the assay [13] (Figure 1A). Median neutralizing antibody levels also increased more than 10-fold in previously infected participants receiving one vaccination with Comirnaty or one vaccination with Vaxzevria (Figure 1B). Overall, Vaxzevria appeared to be inferior in boosting RBD binding and neutralizing antibody titers as compared to Comirnaty, but time since vaccination was longer for Vaxzevria and lower levels are potentially explained by antibody decline (Supplementary Tables S1 and S2).

Third, to evaluate a potential beneficial effect of two doses of Comirnaty in previously infected participants, the antibody levels were compared between participants receiving a single dose of Comirnaty and those receiving a second Comirnaty immunization; second doses were given at a median time interval of 21 days (IQR: 21–21.5) after the first. Although limited by a small number of participants in the group receiving two doses of Comirnaty, individuals with a second dose did not have higher median antibody levels than individuals with one dose; in fact, lower median values were observed. However, these differences were not statistically significant when accounting for multiple testing and potentially due to individuals with two doses being older and vaccinated longer ago (Supplementary Tables S1 and S2).

### 3.2. Antibody Responses Induced by the Various Vaccines among Previously Uninfected Individuals

**Figure 2:**
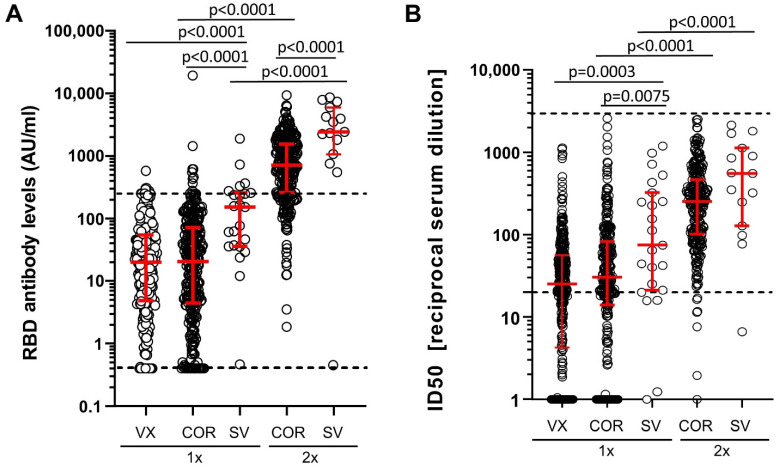
RBD binding and neutralizing antibodies in uninfected participants after vaccination. (**A**) Binding antibody levels to RBD (arbitrary units, AU) and (**B**) neutralizing antibodies (ID50) in N-antibody seronegative participants vaccinated once (1×) or twice (2×) with Vaxzevria (VX), Comirnaty (COR), or Spikevax (SV). The Kruskal–Wallis test including all five groups indicated significant differences for the RBD and neutralizing antibody levels ((**A**) *p* < 0.0001, (**B**) *p* < 0.0001, judged at 5% significance level). Pairwise comparisons were performed to (i) compare antibody response to one vaccination between vaccine types (COR vs. VX, SV vs. VX, SV vs. COR), (ii) compare two versus one vaccination (COR-2x vs. COR-1x, SV-2x vs. SV-1x) and (iii) compare two vaccinations of different vaccine types (SV-2x vs. COR-2x). *p*-values of these pairwise group comparisons with the Mann–Whitney test are shown (judged for significance at a Bonferroni-corrected level of 0.05/6 = 0.008 to account for 6 tests). Dashed lines mark the upper and lower limits of the linear range of the assay.

In participants without prior SARS-CoV-2 infection (N-antibody negative at T1, T3, and if tested T2), median RBD antibody levels after one vaccination with Comirnaty, Vaxzevria, or Spikevax were at least two orders of magnitude lower (Figure 2A) than after one immunization in previously SARS-CoV-2-infected participants (Figure 1A). Median neutralizing antibodies were also at least one order of magnitude lower (Figure 1B and Figure 2B). Even after two immunizations of uninfected participants with Comirnaty of Spikevax, median RBD antibody levels and neutralizing antibody levels were lower than those observed in infected participants after a single immunization with the respective mRNA vaccines (Figure 1 and Figure 2).

With respect to potential differences in the immunogenicity of the different vaccines, we first compared RBD and neutralizing antibody levels after a single immunization. Spikevax induced significantly higher antibody levels than Comirnaty and Vaxzevria (Figure 2A,B). Pairwise comparison of Vaxzevria and Comirnaty revealed slightly higher median neutralizing antibody levels after Comirnaty immunization (Figure 2B), while no difference was observed for median RBD antibody levels (Figure 2A). Of note, individuals with one Spikevax immunization are younger than the group of individuals receiving one dose of Corminaty (median age 56 versus 68 years; Supplementary Tables S3 and S4) and time since vaccination was slightly shorter compared to one VX group individuals (22 versus 34 days; Supplementary Tables S3 and S4).

Second, we compared individuals vaccinated twice with individuals vaccinated once with the respective vaccine. Individuals with two immunizations with Comirnaty and Spikevax had substantially higher median RBD and neutralizing antibody levels than individuals with one immunization with the respective vaccine type (Figure 2A,B; Mann–Whitney test *p* < 0.0001 for both).

Third, median RBD binding antibody levels were also significantly higher after two Spikevax immunizations than after two doses of Comirnaty (Mann–Whitney *p* < 0.0001, Figure 2A). However, the difference in the median neutralizing antibody levels between the two groups did not reach statistical significance (Mann–Whitney *p* = 0.0501, Figure 2B); age and time since vaccination were similar for this group comparison (Supplementary Tables S3 and S4).

Due to the longer time interval recommended between the two Vaxzevria immunizations, only a limited number of samples were available for two doses of Vaxzevria at the T3 assessment of this cohort.

To better understand the variability of measured antibody levels between individuals, we analyzed the impact of age and time since vaccination on RBD binding and virus neutralizing antibody levels (Supplementary Tables S3 and S4). For this, we focused on individuals vaccinated with Comirnaty. First, we evaluated the relationship between age and time since vaccination and found a distinct pattern (Supplementary Figure S1): (i) early on, vaccinated individuals included younger and older age (time since vaccination 50–100 days), (ii) followed by an interim interval where almost exclusively older individuals were vaccinated (30–50 days, 75+ years of age), and (iii) for the period most closely to blood drawn, vaccinated individuals again included all ages. This pattern can bias univariate association estimates of antibodies and age and time since vaccination which prompted us to apply multiple regression analysis allowing for non-linear associations (generalized additive models).

**Figure 3:**
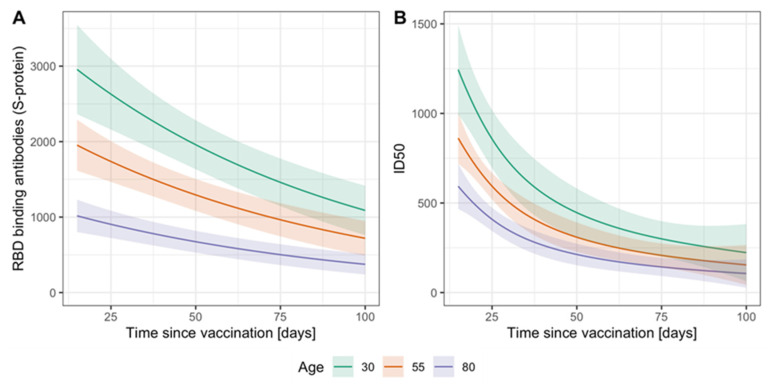
Model-based RBD binding and neutralization antibody levels related to age and time since vaccination. We applied a generalized additive model with assumed log-normal distribution of the antibody levels and non-linear associations with age and time since vaccination on the cross-sectional data from T3. On the example of individuals vaccinated twice with COV, we show predicted levels of (**A**) RBD binding antibodies (arbitrary units AU/mL) and (**B**) neutralizing antibodies (ID50) for the age of 30, 55, and 80 years and over the observed range of days since second vaccination (*n* = 272 and 269, respectively). Shaded regions indicate approximate 95%-prediction intervals (Prediction ± 2×SE).

Multiple regression revealed significant associations of both age and time since vaccination with measured RBD binding antibodies and neutralization titers (Figure 3A,B; *p* < 0.0001 for age and time for both antibody levels). We found a close-to-linear decrease in antibody levels on a log-scale by increasing age and increasing time after the vaccination (Supplementary Figure S2). We also investigated the possibility of a varying functional form for the decrease in antibodies by time since vaccination with changing age based on a bivariate spline surface. We found no evidence for such interaction of the covariates (explained deviance spline surface vs. spline per covariate: 22.3% vs. 23% for RBD binding antibodies and 27.2% vs. 28.3% for neutralizing antibodies). We therefore present results with non-linear effects per covariate. These results indicate that both increased age and increased time since vaccination have an independent decreasing effect on antibody levels.

### 3.3. Breadth of Neutralization after Natural Infection or Vaccination

To determine the breadth of the neutralizing activity after SARS-CoV-2 infection or vaccination, we used a lentiviral pseudotype neutralization assay against the D614G wildtype virus as well as against the Alpha, Beta, and Delta variants of concern (VOC). For this, we selected four random samples of participants (in each, 50% women): one group of participants with SARS-CoV-2 infection and no vaccination (*n* = 30, seropositive at T1, T2, or T3), and 3 groups of uninfected participants vaccinated with 2 doses of Comirnaty (30 participants each by age-groups 18–59, 60–79, 80+ years). In total, the four different neutralizing antibody levels (50% inhibitory dose against WT, Alpha, Beta, and Delta) were evaluated in 120 participants based on blood samples from April 2021.

**Figure 4:**
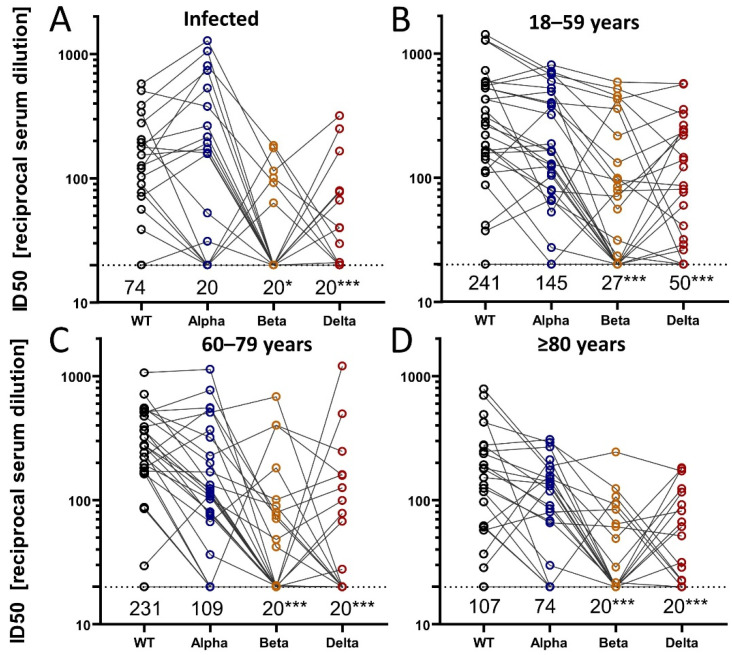
Breadth of neutralization after infection or vaccination. We randomly selected four groups of participants totaling *n* = 120 (30 infected and unvaccinated (**A**), 90 vaccinated twice with Comirnaty from three age groups (**B**–**D**)). The neutralization activity was determined in a lentiviral pseudotype assay using the spike proteins of wild type (D614G) SARS-CoV-2 and the indicated VOCs. Shown are the reciprocal of the 50% inhibitory dilution (ID_50_) of each serum sample by WT or VOCs. Person-specific measurements for WT and VOCs are connected by gray lines. Dashed black lines indicate the lower limit of detection (ID_50_ = 20) and median ID_50_ values per group are stated below this line. We conducted paired tests (Wilcoxon rank-sum test) comparing VOCs with WT and indicate significant differences in the medians by *, *** for *p*-values of <0.01 and <0.0001, respectively.

Among the 30 infected participants, median ID50 values against VOCs were lower compared to wild type (Figure 4A). This was particularly pronounced for Beta and Delta (Wilcoxon test *p* < 0.01). However, a large proportion of individuals were below the lower limit of detection: median ID50 against wildtype was 74 (40% below detection level); median levels for VOCs were below the lower detection limit at 20 (Figure 4A), which indicated that less than 50% of participants had detectable levels of neutralizing antibodies against any VOCs in this pseudotype neutralization assay (Figure 4A).

Among each of the three groups of vaccinated individuals without infection (*n* = 30 each, Figure 4B–D), median ID50 values were lower for VOCs compared to wild type, particularly for Beta and Delta (Wilcoxon test *p* < 0.01).

When comparing the three groups of vaccinated individuals across the different age groups, we found a general tendency of lower ID50 values for older individuals (80+ years) compared to younger for all VOCs. This is in line with the findings of lower RBD binding or neutralizing antibodies from the previous chapter; some of these differences can also be due to differences in time since vaccination as highlighted above.

### 3.4. IgA Response

**Figure 5:**
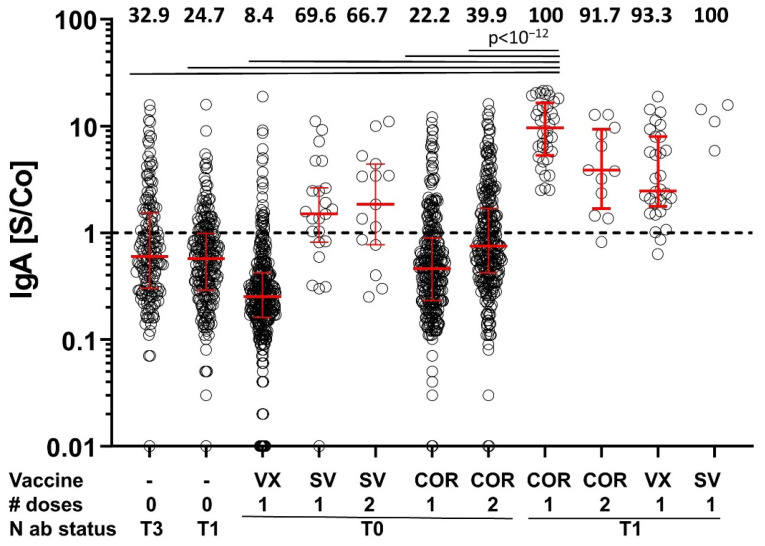
IgA antibody levels. Shown are IgA signal to cut-off ratios (S/Co) for serum samples from the indicated groups of participants. Numbers on top of the graph give the percentage of IgA-positive participants of each group. Fisher’s exact tests were used for pairwise comparison of all groups without correcting for multiple testing (see Supplementary Table S5) and judged at Bonferroni-corrected significance of 0.05/55 = 9.09 × 10^−4^. For clarity reasons, highly significant differences (*p* < 10^−12^) between groups are only shown for comparisons of participants that were N-antibody (ab) seropositive at the June 2020 time point (N ab status: T1) and vaccinated once with Comirnaty (COR). T3: participants negative for N antibodies at the June and November 2020 visits, but positive at the April 2021 visit. N ab status: T0: participants seronegative for N antibodies at all study time points analyzed. VX: Vaxzevria; SV: Spikevax; The dashed line separates IgA seropositive from seronegative samples. Supplementary Tables S5 and S6 provide *p*-values of all pairwise comparisons as well as number of participants, basic demographic characteristics and the median time interval between the last immunization and blood sampling. # doses indicates the number of applied doses.

To further explore potential differences in the antibody responses to infection without and with vaccination, IgA-specific antibody responses against RBD were also determined in samples collected in April 2021 using our in-house RBD ELISA. Unvaccinated participants that were N-antibody seropositive in June 2020 and those that seroconverted for N antibodies between November 2020 and April 2021 (recently infected) were predominantly IgA negative (Figure 5). Most participants that were N-antibody seronegative at all three study time points and vaccinated once with Vaxzevria or once or twice with Comirnaty did not develop detectable IgA antibody levels to the RBD, either (Figure 5). However, after one or two immunizations with Spikevax, more than half of the vaccinees had detectable levels of IgA antibodies to RBD. Interestingly, participants that were already N-antibody seropositive in June 2020 (infection >9 months ago) and subsequently immunized once with any of the COVID-19 vaccines showed elevated IgA levels for all vaccine types (Figure 5, Supplementary Tables S5 and S6).

When comparing the percentage of IgA positives of uninfected individuals vaccinated twice with COR to one-time vaccinated individuals with infection >9 months ago (39.9% and 100%, respectively), we found a significant difference (exact Fisher’s test *p* < 10^−20^, Supplementary Table S5). This indicates that a prior SARS-CoV-2 infection primes for stronger IgA responses than one immunization with Comirnaty, which is in line with previous work [21].

## 4. Discussion

Early on, binding and neutralizing antibody levels have been proposed as correlates of protection after vaccination with COVID-19 vaccines [22]. Recently, Gilbert et al. indeed observed a correlation of RBD binding /neutralizing antibody levels four weeks after the second Spikevax immunization with vaccine efficacy during a 100 day period after the second immunization [23]. Although proof that this correlation can be extended to other vaccines or antibody levels determined at later time points after immunization is lacking, recommendations on immunization practices have partially been based on immunogenicity data, since efficacy or effectiveness data were not available.

The need for national booster immunization campaigns has been widely discussed during summer 2021 and weighted against prioritization of available vaccine doses for resource-poor settings in which primary immunization is still lacking behind. Tailored recommendations for booster immunizations limited to particular age groups or less effective vaccines may help to reconcile these divergent requests. Herein, we could show a significant decrease in antibody levels dependent on both age and time since vaccination, rationalizing a prioritization scheme based on age for 3rd vaccinations. The latter becomes particularly important with the emergence of Omicron, for which a close to 40-fold decrease in in vitro neutralization in sera even from recently fully vaccinated individuals has been reported [24]. Interestingly, a total of three antigen exposures by either infection or vaccination induced better neutralizing activity against all variants of concern [25]. However, further booster immunizations for all or particular age groups may become necessary as waning protection has also been observed after the 1st booster immunization [26]. Expanded booster campaigns should be accompanied by the quest to expand global manufacturing capacities and timely adoption of vaccine antigens.

The extent of antibody levels after infection compared to vaccination and a combination is still a current debate. For vaccinated individuals, we document an independent relationship of declining antibody response by older age and longer time since vaccination. Furthermore, the changing recommendations of different vaccines for different age groups provide a relationship of time since vaccination and age to vaccine type. These aspects render group comparisons inherently complex, between different vaccines as well as between vaccination and infection. Nevertheless, our data show substantially higher RBD binding and neutralizing antibody responses to a single vaccination after infection compared to a single vaccination without infection. It was reported that vaccination provided better protection from COVID-19 than a past infection [27,28]. However, two other publications indicate that protection from (re-)infection with the Delta variant after a first SARS-CoV-2 infection may be stronger than the protection conferred by two Comirnaty immunizations [29,30]. These data are supported by findings from Wang and colleagues reporting that, in the absence of vaccination, antibody reactivity to the receptor-binding domain (RBD) of SARS-CoV-2, neutralizing activity, and the number of RBD-specific memory B cells remain relatively stable between 6 and 12 months after infection [31]. Further evidence suggests that vaccination following natural infection increases all components of the humoral response and yields serum neutralizing activities against variants of concern (VOC) [32,33,34]. However, additional studies are needed to draw firm conclusions on the degree and duration of protection from re-infection after a first SARS-CoV-2 infection.

The analysis of serum IgA levels after SARS-CoV-2 infection combined with vaccination reveals for the first time that SARS-CoV-2 infection primes for a stronger IgA response after a single immunization with any of the three COVID-19 vaccines, than either two vaccinations in previously uninfected participants or a SARS-CoV-2 infection by itself. The higher proportion of IgA positives in infected individuals after one immunization parallels the increased effectiveness observed after a single dose of Comirnaty in previously infected individuals. However, it remains to be determined whether IgA levels are indeed a good correlate of protection in individuals vaccinated after a previous SARS-CoV-2 infection.

As reported previously, we also observed reduced immunogenicity of Comirnaty in the elderly consistent with the evidence for a higher risk for breakthrough infections in this age group and the quest for a booster vaccination [35]. Comparisons of differential effectiveness by different vaccines need to take age and time since vaccination into account. We did not observe a significant difference in S antibody responses after two doses of Comirnaty between male and female participants of our study. However, an in-depth analysis is still needed to adjust for age and time since vaccination.

Importantly, we document the decline in neutralization antibodies for the Beta and Delta VOCs across all age groups for vaccinated as well as infected individuals. This highlights the relevance of VOCs in future handling of the pandemic.

## 5. Conclusions

In conclusion, we found that RBD binding and neutralizing antibody levels after two mRNA immunizations of previously uninfected individuals are higher than those raised by SARS-CoV-2 infection, but lower than titers raised by SARS-CoV-2 infection combined with one vaccination. Furthermore, we found a clear dependency of vaccine-elicited antibodies on age and time since vaccination. Finally, we could show increased IgA responses for previously infected and vaccinated participants in comparison to infection or vaccination by itself.

## Data Availability

All authors declare that data and materials will be made available according to the guidelines of the journal.

## Supplementary Materials

The following are available online at https://www.mdpi.com/article/10.3390/vaccines10020324/s1: Table S1: A Composition of study population: Infected unvaccinated versus infected and vaccinated individuals tested for Spike-RBD binding antibodies (Supplement to Figure 1A). Table S2: Composition of study population: Infected unvaccinated versus infected and vaccinated individuals tested for neutralization (Supplement to Figure 1B). Table S3: A Composition of study population: non-infected individuals immunized with different vaccines, tested for Spike-RBD binding antibodies (Supplement to Figure 2A). Table S4: Composition of study population: non-infected individuals immunized with different vaccines, tested for neutralizing antibodies (Supplement to Figure 2B). Table S5: *p*-values of pairwise comparisons with two-sided Fisher’s exact test. Table S6: Composition of study population: IgA Spike-RBD binding antibodies in infected and vaccinated individuals (Supplement to Figure 5). Figure S1: Scatterplot of the observed time since full vaccination and age of participants in the subset of individuals that were vaccinated twice with the Comirnaty vaccine. Figure S2: Estimated non-linear associations of time since vaccination and age at BL with RBD binding antibody levels and neutralizing antibodies.
