# Supplementary material for: Comparative Immunogenicity of COVID-19 Vaccines in a Population-Based Cohort Study with SARS-CoV-2-Infected and Uninfected Participants
Source: Vaccines (Basel). 2022 Feb 18;10(2):324. doi: 10.3390/vaccines10020324 (PMC8875516; doi:10.3390/vaccines10020324)
Supplement: Supplementary file 1 [file vaccines-10-00324-s001.zip › vaccines-1574576-supplementary.pdf]

vaccines
MDPI

Supplemental Figures

Supplemental Figure S1

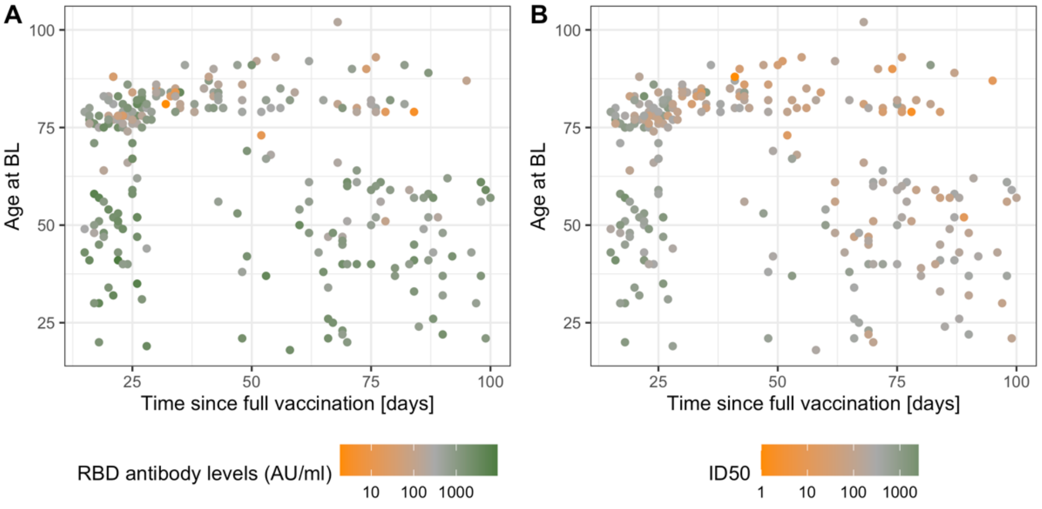

Supplementary Figure S1: Scatterplot of the observed time since full vaccination and age of participants in the subset of individuals that were vaccinated twice with the Comirnaty vaccine at least 14 days before blood draw. Panel A shows the measured RBD binding antibody levels of the 272 individuals, panel B the measured ID50 levels of 269 individuals in color.

Supplemental Figure S2

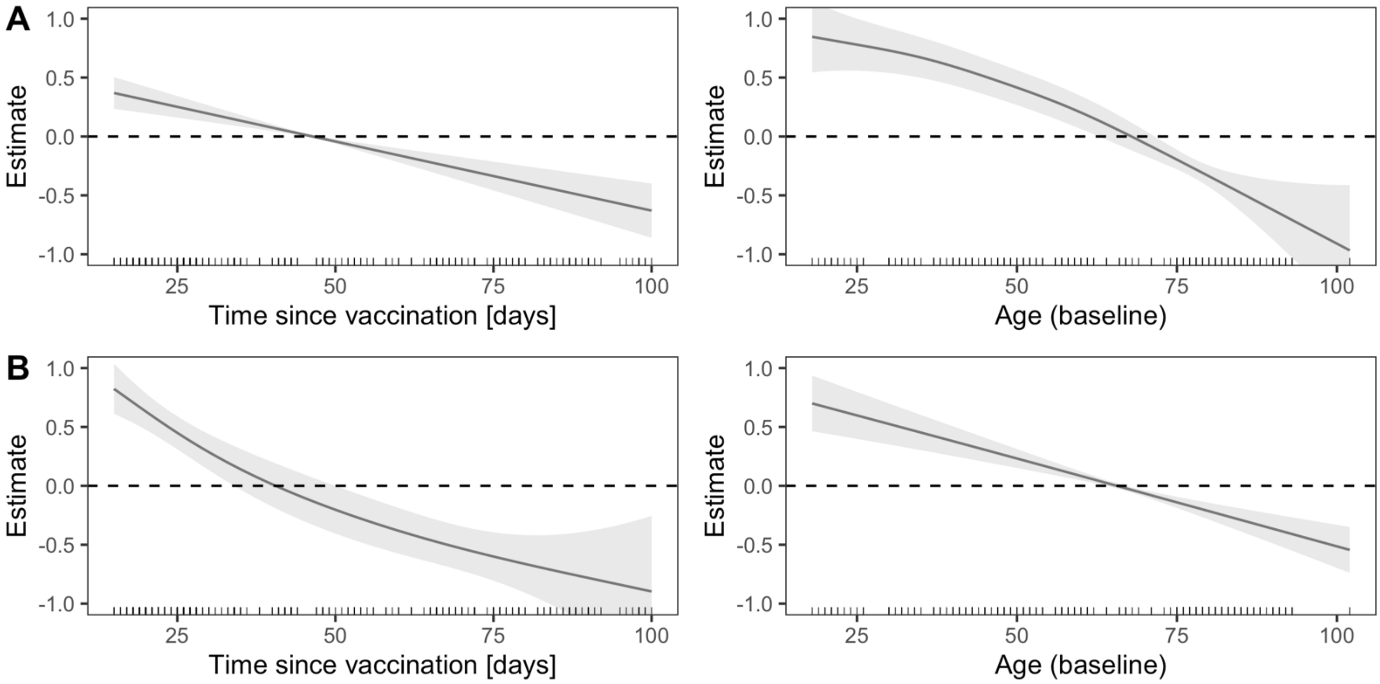

Supplementary Figure S2: Estimated non-linear associations of time since vaccination and age at BL with RBD binding antibody levels (Panel A) and neutralizing antibodies (ID 50, Panel B). Shown the estimated non-linear effects and 95%-confidence intervals.

Supplemental Tables

Supplemental Table S1. Composition of study population: Infected unvaccinated versus infected and vaccinated individuals tested for Spike-RBD binding antibodies. (Supplement to Fig. 1A)

N ab status	T3	T1				
Doses, vaccine	0	0	1, VX	1, COR	1, SV	2, COR
N	164	227	30	36	4	12
Sex, female / male	90/74	121/106	17/13	15/21	0/4	10/2
AU/ml, median (IQR)	73 (31-303)	171 (60.8-474)	14185 (6471-23234)	42967 (25114-57335)	114049 (70011-148592)	15324 (4053-56841)
Age in years, median (IQR)	46 (28-56)	49 (31.5-58)	63.5 (46.75-73.5)	66.5 (50-68.25)	65.5 (48-75,25)	56.5 (48-78,5)
Days since last immunization Median (IQR)	n.a.	n.a.	44 (27.75-52.75)	23 (17-29.5)	26,5 (24-27)	54 (24.75-78.75)

Supplemental Table S2. Composition of study population: Infected unvaccinated versus infected and vaccinated individuals tested for neutralization (Supplement to Fig. 1B)

N ab status	T3	T1				
Doses, vaccine	0	0	1, VX	1, COR	1, SV	2, COR
N	164	226	28	36	4	12
Sex, female / male	90/74	120/106	15/13	15/21	0/4	10/2
ID50, median (IQR)	140 (63-313)	72 (34-146)	1013 (639-1898)	2558 (1377-3001)	3001 (2971,75-3001)	1591 (343-2712)
Age in years, median (IQR)	46 (28-56)	49 (31.25-57.75)	63.5 (48.25-74.5)	66.5 (50-68.25)	65.5 (48-75,25)	56.5 (48-78,5)
Days since last immunization Median (IQR)	n.a.	n.a.	45 (29.25-53)	23 (17-29.5)	26,5 (24-27)	54 (24.75-78.75)

Supplemental Table S3. Composition of study population: non-infected individuals immunized with different vaccines, tested for Spike-RBD binding antibodies (Supplement to Fig. 2A)

Doses	1 immunization			2 immunizations	
Vaccine	VX	COR	SV	COR	SV
N=	321	287	23	271	15

Sex, female / male	179/142	135/152	12/11	163/108	10/5
AU/ml, median (IQR)	20 (4.9-53.5)	20.4 (4.4-71.1)	152 (36.3-251)	713 (262-1524)	2416 (1433-5067)
Age in years, median (IQR)	57 (45-71)	68 (57-71)	56 (48-68,5)	76 (50-81)	76 (55.5-83)
Days since last immunization Median (IQR)	34 (25-48)	21 (18-25)	22 (19.5-26)	38 (23.5-69.5)	48 (25-70.5)

Supplemental Table S4. Composition of study population: non-infected individuals immunized with different vaccines, tested for.neutralizing antibodies (Supplement to Fig. 2B)

Doses	1 immunization			2 immunizations	
Vaccine	VX	COR	SV	COR	SV
N=	319	285	23	268	15
Sex, female / male	178/141	133/152	12/11	160/108	10/5
ID50, median (IQR)	25.3 (4.3-55.8)	30.3 (14.1-81.8)	74.9 (23.2-288)	253 (103-463)	554 (188-1016)
Age in years, median (IQR)	57 (45-70.5)	68 (57-71)	56 (48-68,5)	76 (50-81)	76 (55.5-83)
Days since last immunization Median (IQR)	34 (25-48)	21 (18-25)	22 (19.5-26)	39 (23-70)	48 (25-70.5)

Supplemental Table S5. P-values of pairwise comparisons with two-sided Fisher´s exact test (Supplement for Fig. 5).

	**0, T1**	**1, VX, T0**	**1, SV, T0**	**2, SV, T0**	**1,COR, T0**	**2,COR, T0**	**1,COR, T1**	**2,COR T1**	**1, VX, T1**
0, T3	0.087	$<10^{-10}$	0.001	0.021	0.014	0.154	$<10^{-14}$	$<10^{-4}$	$<10^{-9}$
0, T1		$<10^{-6}$	$<10^{-4}$	0.001	0.465	$<10^{-3}$	$<10^{-18}$	$<10^{-5}$	$<10^{-12}$
1, VX, T0			$<10^{-10}$	$<10^{-6}$	$<10^{-5}$	$<10^{-19}$	$<10^{-31}$	$<10^{-9}$	$<10^{-23}$
1, SV, T0				1	$<10^{-5}$	0.008	$<10^{-3}$	0.216	0.031
2, SV, T0					$<10^{-3}$	0.057	0.001	0.182	0.032
1,COR, T0						$<10^{-5}$	$<10^{-20}$	$<10^{-5}$	$<10^{-14}$
2,COR, T0							$<10^{-12}$	$<10^{-3}$	$<10^{-7}$
1,COR, T1								0.250	0.203
2,COR T1									1

T1, T3, VX, SV, COR as in Figure legend 5. T0: participants seronegative for N antibodies at all study time points.

Supplemental Table S6. Composition of study population: IgA Spike-RBD binding antibodies in infected and vaccinated individuals (Supplement for Fig. 5)

N ab status	T3	T1	N antibody negative						T1			
Doses, vaccine	0	0	1, VX	1, SV	2, SV	1, COR	2, COR	1, COR	2, COR	1, VX	1, SV
N	164	227	323	23	15	288	271	36	12	30	4
Sex, female / male	90/74	121/106	180/143	12/11	10/5	135/153	163/108	15/21	10/2	17/13	0/4
# IgA [S/Co] >1 / # IgA [S/Co] ≤1	54/110	56/171	27/296	16/7	10/5	64/228	108/163	36/0	11/1	28/2	0/4
IgA [S/Co] median (IQR)	0.60 (0.30-1.54)	0.57 (0.29-0.98)	0.25 (0.16-0.42)	1.50 (0.82-2.55)	1.85 (0.81-3.93)	0.46 (0.24-0.88)	0.75 (0.42-1.65)	9.62 (5.61-14.3)	3.83 (2.13-8.69)	2.47 (1.85-7.44)	12.6 (9.72-14.6)
Age in years, median (IQR)	46 (28-56)	49 (31.5-58)	57 (45-71)	56 (48-68,5)	76 (55.5-83)	68 (57-71)	76 (50-81)	66.5 (50-68.25)	56.5 (48-78,5)	63.5 (46.75-73.5)	65.5 (48-75,25)
Days since last immunization Median (IQR)	n.a.	n.a.	34 (25-48)	22 (19.5-26)	48 (25-70.5)	21 (18-25)	38 (23.5-69.5)	23 (17-29.5)	54 (24.75-78.75)	44 (27.75-52.75)	26,5 (24-27)
